# Shear Strengthening of RC Beams Using Prestressed Near-Surface Mounted Bars Reducing the Probability of Construction Failure Risk

**DOI:** 10.3390/ma17235701

**Published:** 2024-11-21

**Authors:** Sabry Fayed, Mohamed Ghalla, Jong Wan Hu, Ehab A. Mlybari, Abdullah Albogami, Saad A. Yehia

**Affiliations:** 1 Civil Engineering Department, Faculty of Engineering, Kafrelsheikh University, Kafr El-Sheikh 33516, Egypt; sabry_fayed@eng.kfs.edu.eg (S.F.); mohamed_shabaan@eng.kfs.edu.eg (M.G.); 2 Department of Civil and Environmental Engineering, Incheon National University, Incheon 22012, Republic of Korea; 3 Incheon Disaster Prevention Research Center, Incheon National University, Incheon 22012, Republic of Korea; 4 Department of Civil Engineering, College of Engineering and Architecture, Umm Al-Qura University, Makkah 24382, Saudi Arabia; eamlybari@uqu.edu.sa; 5 Department of Civil Engineering, Faculty of Engineering, Al-Baha University, Al-Baha 65779, Saudi Arabia; albaqmi.a@bu.edu.sa; 6 Department of Civil Engineering, Higher Institute of Engineering and Technology, Kafr El-Sheikh 35516, Egypt; saad_yehia@kfs-hiet.edu.eg

**Keywords:** RC beams, NSM, shear strengthening, grooves, drilled holes, prestressing, construction failure, risk mitigation, sustainability

## Abstract

In this study, shear-critical reinforced concrete (RC) beams were strengthened by combining the prestressing and near-surface mounted (NSM) rods approaches. The potential danger of failure in such RC beams is a substantial concern as it is considered a potential threat. This study addresses its careful mitigation through experimental identification and numerical analysis to enhance the safety and sustainability of buildings by reducing the probability of failure risk for these RC beams. Nine of the ten RC beams that were tested had strengthened, and one had not. Internal prestressing (IP) within the beam body, external prestressing NSM (PNSM), internal embedment (IE) inside the beam with or without prestressing, and NSM are the strengthening technologies that were employed. The range of the extra shear reinforcement ratios (μs) was 0.87% to 1.60%. We investigated how strengthened beams behaved structurally in terms of the cracking load, ultimate load, load–deflection response, ultimate deflection, and stiffness. The insertion of five pairs of PNSM rods (μs = 1.45%) and five pairs of IP rods (μs = 1.6%), respectively, increased the beams’ shear capacity by 57.8% and 70.4%. Shear capacity increased by 23.2% when three pairs of IE rods (μs = 1.02%) were installed. The prestressing location had an impact on shear capacity, with the interior case surpassing the external one. Compared to the control, the stiffness of the strengthened beams rose by 20%, 82%, and 84.4% when three, four, or five pairs of internal prestressing rods were added. A formula is proposed to calculate the shear capacity of all beams strengthened using various methods.

## 1. Introduction

Careful consideration and effective management are crucial to ensure safe and sustainable building, mitigating the potential existence of additional risks that could lead to construction failure. Accordingly, in recent years, strengthening structural parts has drawn a lot of attention from researchers. Overall, attacks by nature, changes in the initial application that result in a rise in the loads applied, or structural enhancing to adhere to and employ the updated set of design codes and regulations require strengthening of the supporting structures of reinforced concrete (RC) structures, particularly beams [1,2]. Existing reinforced beams underwent strengthening procedures to increase their shear and flexural capabilities [3,4]. Externally bonded reinforcement (EBR) is one of the key techniques utilized in RC beam restoration [4,5]. In recent years, construction materials for exterior reinforcement have included fiber-reinforced polymer (FRP), reinforced steel, and steel plates [3,5]. Because of its excellent tensile resistance and high toughness, the employment of steel reinforcing technology to increase the shear resistance of RC beams was examined. The most realistic methods of strengthening incorporate it through portions, the near-surface strategy, and EBR. The past few years have seen an increase in the quantity of studies on the strengthening of RC beams using externally bonded (EB) and near-surface mounted (NSM) methods [6,7,8,9,10,11,12,13,14,15,16,17]. Compared to the EB reinforcing technique, the NSM system offers a number of advantages, such as less surface setup, robust bond power, enhanced design, and the defense of strengthening construction materials against environmental breakdown. Based on the findings of trials that were documented in the literature [18,19], the failure mechanisms of the NSM approach are divided into two categories: (I) concrete cover division, which happens close to the end of an NSM bar and expands as loading rises; (II) middle crack-induced debond, which usually starts at a middle crack at the surface of a strengthened beam.

Studies [20,21] have demonstrated that RC components reinforced with outwardly attached FRP plates are susceptible to ageing and can suffer from FRP debonding at relatively low stresses. NSM is a novel strengthening approach that uses fiber-reinforced polymer (FRP) inserted into slots made in the concrete covering of RC structures, which was employed to get around the drawbacks of the EB method. Numerous studies have looked at the application of NSM in the shear, flexure, and compression strengthening of reinforced concrete structures [22,23,24,25,26,27,28,29,30,31,32,33,34]. After taking into account a variety of factors, including the quantity, orientation, and kind of FRP, the diameters of the grooves, the location and dimension of the NSM, and the kind of adhesive, researchers discovered that the use of NSM improves the flexural and shear resistance of RC components. Higher angles of inclination and shorter distances of CFRP result in greater enhancements in the shear resistance of NSM CFRP-reinforced samples, according to research [35,36,37]. Furthermore, a too-high CFRP percentage may cause the failure mode to change from CFRP separating to web concrete cover division, which might cause the sample to fail earlier than expected [38,39,40]. In order to compare them with the rods affixed on the beam surface, the rebars in the present investigation will be embedded in the center of the beam region.

According to engineering standards, non-prestressed CFRP reinforcement is capable of supporting the stress brought on by outside forces, which will only slightly increase the structure’s stiffness and resistance to cracking. On the contrary, CFRP is initially subjected to strain or tension by the prestressed CFRP reinforcing process [41,42]. This method can completely use the excellent tensile resistance of CFRP, counter tension stresses, and avoid or decrease possible cracking. Approaches for prestressed EB CFRP shear strengthening were examined in earlier research. Using techniques like self-locking tightening [43,44], rotating machines [45], and pulling CFRP loops [46,47], they introduced prestress to CFRP buckles or strips on the web. This method enhanced the samples’ shear strength and successfully prevented the formation of shear fractures. The initial prestress intensity and CFRP distance were two parameters that had a big impact on the strengthening effectiveness. Yet, in order to sustain the prestress, prestressed EB CFRP needs expensive, corrosion-prone permanent metal anchors linked to the reinforced structure. The anchors in the prestressed NSM CFRP technology can be removed after the CFRP has bonded to the concrete because a robust bonding performance allows the prestress to be retained. The expense of this method is decreased by the replaceable anchoring and tensioning tools. Because of this benefit, prestressed NSM CFRP is thoroughly investigated for concrete beam flexural reinforcement. At specific prestress stages, it can increase the ability to deform samples while enhancing the final load bearing and cracking of RC beams [48,49]. As a result, this is the main aim of the current study.

The majority of previous research employed NSM steel rods and CFRP rods bonded on the sidewalls of shear-critical RC beams, according to the background survey mentioned above. In order to make use of the benefits of the thick concrete covering around the embedded rods, this study installs steel rods inside the beam web, going above and beyond the existing state of the art. The structural capability of beams reinforced with NSM-AA rebars was tested using ten beams [50]. The control criteria included the kind of bonding agent, end anchoring of aluminum alloy bars, bonded length of aluminum alloy bars, and reinforcing ratio of aluminum alloy (AA) bars. The ultimate capacity of the NSM AA bars-reinforced RC beams was much higher than that of the control sample, ranging from 35% to 120%. Two control beams strengthened with plain steel bars and nine naturally occurring concrete beams reinforced with AA rebars were constructed and tested to fail under four bends [51]. NSM 7075 AA bars were used to create and enhance five beam prototypes [52]. The test results showed that the application of the NSM AA technology increased the ability to bend of the reinforced beams by 35% in comparison to the control beam. Mahmoud [7] examined how beams reinforced using the NSM method behaved when flexed until they failed. The results of the studies demonstrate that NSM components produce high-quality outcomes.

**Figure 1:**
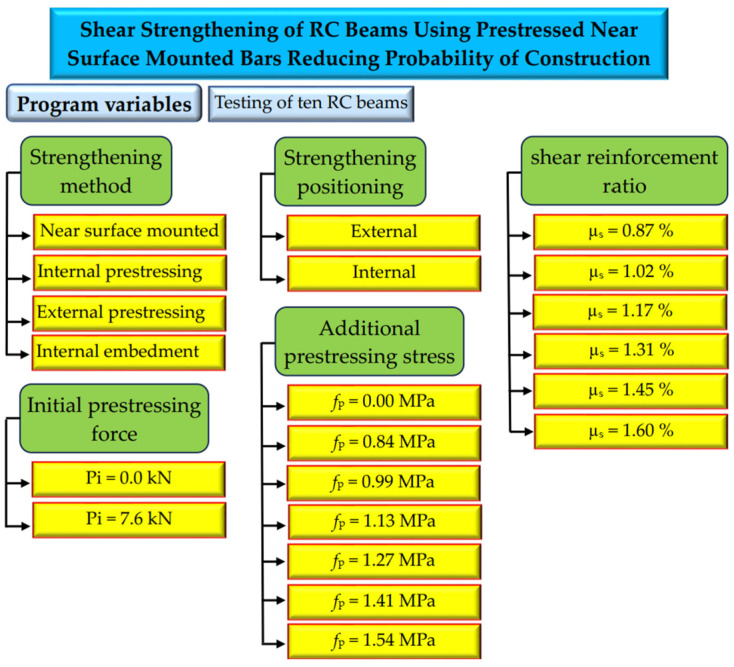
Workflow diagram for the experimental program.

In this investigation, the applicability of the prestressed NSM technology for shear strengthening in RC beams is conducted. To concentrate on issues like shear fractures and high beam deflection, an NSM rod is prestressed and inserted into concrete slots on both sides of the beam web. In order to prevent issues with the NSM approach, internal prestressed steel rods were also inserted inside the beam body. To confirm the efficacy of the used procedures, prestressed NSM shear-strengthened RC beams underwent bending loading tests. This research also indicates the possible failure mechanisms that reinforced beams may experience. Analysis is conducted on the impacts on the shear crack load, failure modes, deformation, stiffness, and ultimate shear capacity of the prestressing NSM level, extra shear reinforcement ratios, placement of insertion rods, and existing prestressing or removal of it. In conclusion, the research suggests a formula utilizing the ACI to forecast the shear strength of strengthened beams and assesses the precision of the forecast. The experimental program tested herein can be summarized in Figure 1.

## 2. Experimental Testing Program

### 2.1. Materials

#### 2.1.1. Concrete

A standard concrete mix was used to cast ten test beams. The components of the concrete mix were as follows: water, graded crushed basalt limestone with an ideal diameter of 12 mm as a coarse aggregate, sand as a fine aggregate, and Portland cement with a 42.5-degree (Table 1). The range of particle sizes for basalt and sand was 2.36 to 12.5 mm and 0.075 to 2.36 mm, respectively. Cement, sand, and basalt had respective weights of 1200, 1300, and 1200 kg/m^3^. Cement, sand, and basalt had specific weights of 3.1, 2.7, and 2.4, respectively. The concrete mix’s compressive property was calibrated to be 25 MPa.

#### 2.1.2. Internal Reinforcement

**Figure 2:**
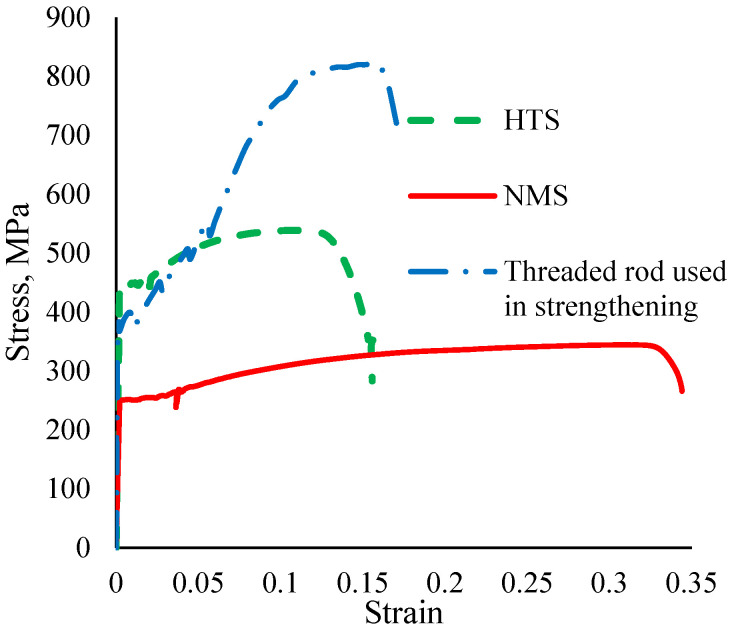
Tensile stress–strain relations of the steel rebars used in the current investigation.

Both normal mild steel (NMS) and high-tensile steel (HTS) were used as steel rebars for the internal reinforcement of the beams. HTS was an object with surface ribs, while NMS was a crossbar with a smooth surface. To ascertain the employed steel bars’ mechanical characteristics, tension tests were carried out. The stress–strain profiles of the different bars are shown in Figure 2. For NMS and HTS, the yield stress was 251 MPa and 439 MPa, respectively. Furthermore, NMS and HTS had tensile strengths of 345 MPa and 530 MPa, respectively. NMS and HTS had an elongation of 33% and 12%, respectively.

#### 2.1.3. Strengthening Tendons and Plates

The surface of the threatened rods included helical ribs that served as a prestressing tendon. Medium carbon steel was used to create the rod. These rods were stainless steel as well. The rods had a 10 mm diameter. The entire length of the rod was threaded. The rod was connected by two nuts, one at each end. The results of rod testing under tension showed that the rod’s final tensile strength was 815 MPa, and its yield strength was 390 MPa (Figure 2). The elongation of this rod was 15%. The beams were strengthened by the use of rigid steel plates with defined areas.

#### 2.1.4. Strengthening Epoxy

The epoxy employed in this investigation is called Sikadur 31CF [53]. This product is described as an extremely strong epoxy glue that is utilized to fuse the exterior plates and rebar to the old concrete. The benefits of Sikadur 31CF include its high capacity, ease of application, lack of priming material requirement, non-slump characteristics, and strong resistant to chemicals. The item has an initial cure duration of thirty minutes and an operation time of seven days. Part A and Part B were the two elements that made up the Sikadur 31CF. The components of Parts A and B were combined and had the same hue. Neither wet concrete nor skin surfaces should be treated with the product. The interior of any holes or grooves in the concrete was thoroughly scrubbed. The approximate thickness of the Sikadur 31CF was 1 to 1.5 mm.

### 2.2. Specimen Details

**Figure 3:**
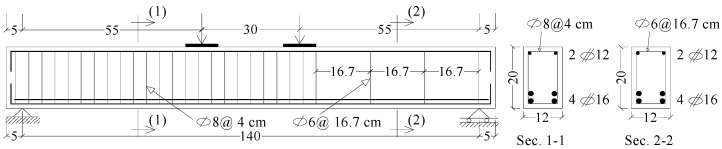
Reinforcement and dimension details of the test beams, dim in cm.

Ten RC beams with identical dimensions and internal reinforcement were mixed, produced, and tested under flexure until failure (Figure 3). The cross-sectional specifications of the beams were 120 mm in width and 200 mm in height, with a loaded span of 1400 mm. Tension or compression internal reinforcement has a 15 mm transparent concrete cover on all sides. The outlines of the beam design stated in [54,55,56] were used in this study. To prevent flexure failure, four HTS bars with a 16 mm diameter were used to strengthen the beams at the tension side. Additionally, to prevent concrete crushing under heavy loads, two 12 mm-diameter HTS bars were added to the beams’ compression side reinforcement. The central section, which measures 300 mm, and the two equal shear spans, each spanning 550 mm, make up the loaded span of the beam. The middle part (300 mm) and the left shear part are well reinforced in the shear to prevent the collapse of the shear in the left span of the shear and force the collapse to occur in the shear in the right span, which we will externally support with different methods. Regarding the stirrups, 8 mm-diameter NMS stirrups were used at a distance of 40 mm at the middle part and the left shear part while 6 mm-diameter NMS stirrups were used at a distance of 167 mm at the right shear part. The outside-dimension and internal reinforcement used in this study were similar with those used in some previous works [57,58,59].

The internal shear reinforcement ratio ($\mu_{si}$) was estimated by the following equation.

(1) $$\mu_{si}=\frac{2A_{s}}{bS}$$

where $A_{s}$ is the cross-sectional area of the stirrup branch which having a 6 mm diameter and 8 mm in the strengthened and unstrengthened shear span. b is the beam breadth (120 mm). *S* is the spacing of the stirrups which was 167 mm and 40 mm in the strengthened and unstrengthened shear span. The shear reinforcement ratio ($\mu_{si}$) of the strengthened and unstrengthened shear span was 0.28% and 2.08%, respectively.

### 2.3. Testing Setup and Measurements

**Figure 4:**
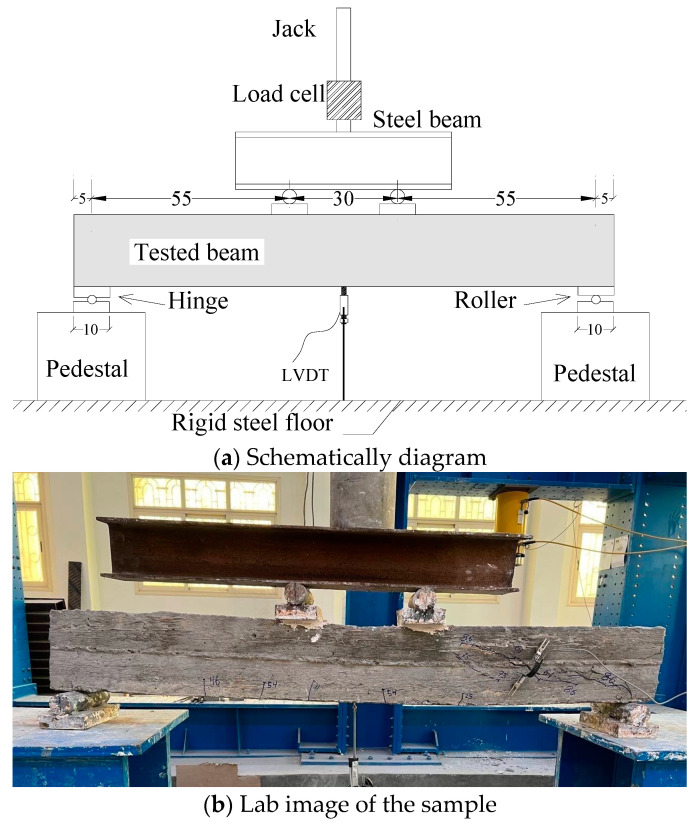
Test setup (cm).

As seen in Figure 4, the examined beams received loads under 4-point flexural testing. The hydraulic load was transmitted into two loading sites using a robust steel loading beam. To prevent crushing of the concrete and securely transmit the applied loads, two steel plates measuring 10 by 10 by 3 cm were placed between the steel beam and the tested beam. The hydraulic jack had a 100 kN capability. In that configuration, a hinge served as the first supporter and a roller served as the additional one. To gauge the mid-span deflection, a linear variable differential transducer (LVDT) was mounted at the center of the beam span. The test setup used in this study were similar with that used in some previous works [57,58,59].

### 2.4. Strengthening

In addition to one unstrengthened beam (B0), nine beams were strengthened, four of which were externally strengthened by gluing a steel rod to the beam sides and five of which were internally strengthened by the embedment of steel rod inside the beam web. In all beams, the strengthening process is conducted on the right shear span measuring 550 mm. The strengthening methods carried out are near-surface mounted (NSM), external prestressing NSM (PNSM), internal prestressing (IP) inside the beam, and internal embedment (IE) inside the beam without prestressing.

**Figure 5:**
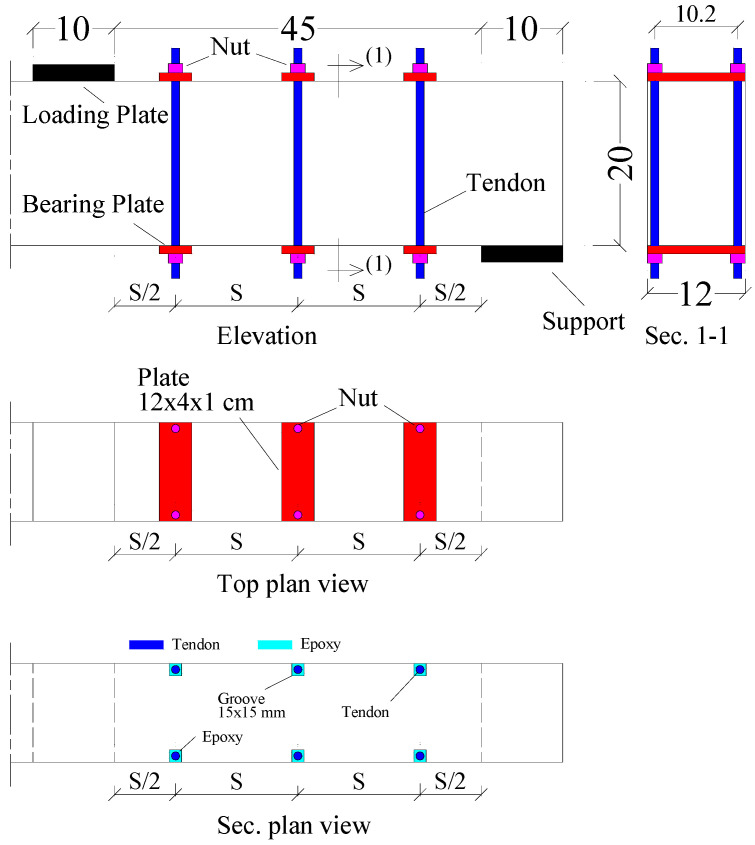

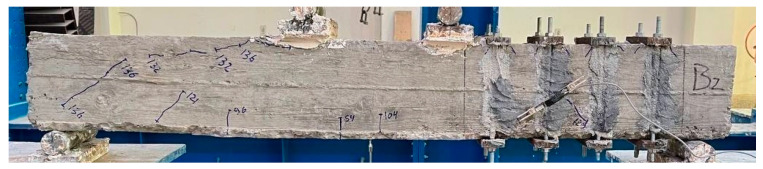
Configuration of external prestressing NSM conducted in three beams: PN3, PN4, and PN5 (cm).

Figure 5 shows the external prestressing NSM conducted in three beams, PN3, PN4, and PN5. Slots are cut on both sides of the beam at the full height of the beam. The cross-sectional area of the slot is 15 × 15 mm. The groove is cleaned well and then partially filled with epoxy. The rods used in strengthening are installed inside the slots, the steel plates are installed inside the rods, and the nuts are partially tightened. After that, all grooves are completely filled with epoxy. Then, the nuts are tightened until the initial prestressing force ($P_{i}$) reaches the required level. A tensile force was generated in the threaded rods by tightening the nuts until the force on the rods reached 25% of its yield strain. A strain gauge glued to the rod was used to read the tensile strain. In the beams PN3, PN4, and PN5, the number of prestressing pair tendons was 3, 4, and 5, respectively (Table 2). Additionally, the spacing (*S*) between the prestressing pair tendons was 150, 112, and 90 mm, respectively, in the beams PN3, PN4, and PN5. The added shear reinforcement ratio due to the strengthening process ($\mu_{s}$) was estimated by the following equation and is listed in Table 2:

(2) $$\mu_{s}=\frac{2A_{sv}}{bS}$$

where $A_{sv}$ is the cross-sectional area of the strengthening rod having a 10 mm diameter, while b is the beam breadth (120 mm).

Applying external pre-tensioning on the strengthening rods caused additional normal stress ($f_{p}$) perpendicular to the beam web plan. This additional stress can be calculated by Equation (3).

(3) $$f_{p}=\frac{2P_{i}}{bS}$$

where $P_{i}$ is the applied prestressing force in each strengthening tendon, which equals $0.25f_{y}A_{sv}$.

NSM rods without prestressing were applied in the beam N4. The configuration drawn in Figure 5 was used in the beam N4 with the exception of the tightening nuts. In this beam, the number and spacing of prestressing pair tendons was 4 and 112 mm, respectively. Also, the µs was 1.17%, while the $f_{p}$ was zero due to absence of prestressing force.

In five beams, IP3, IP4, IP5, IE3, and IE3*, vertical round grooves of 14 mm in diameter were drilled at full depth inside the beam section. This method is distinguished by two things. First, burying the rods inside the beam’s web achieves a greater thickness of the concrete around the buried rod compared to the NSM rod glued to the beam surface. Secondarily, the inclination angle of each strengthening rod pair with the axis of the beam was 45 degrees, which led to a reduction in the distance of the strengthening rods and thus resisted shearing better than the NSM rods.

**Figure 6:**
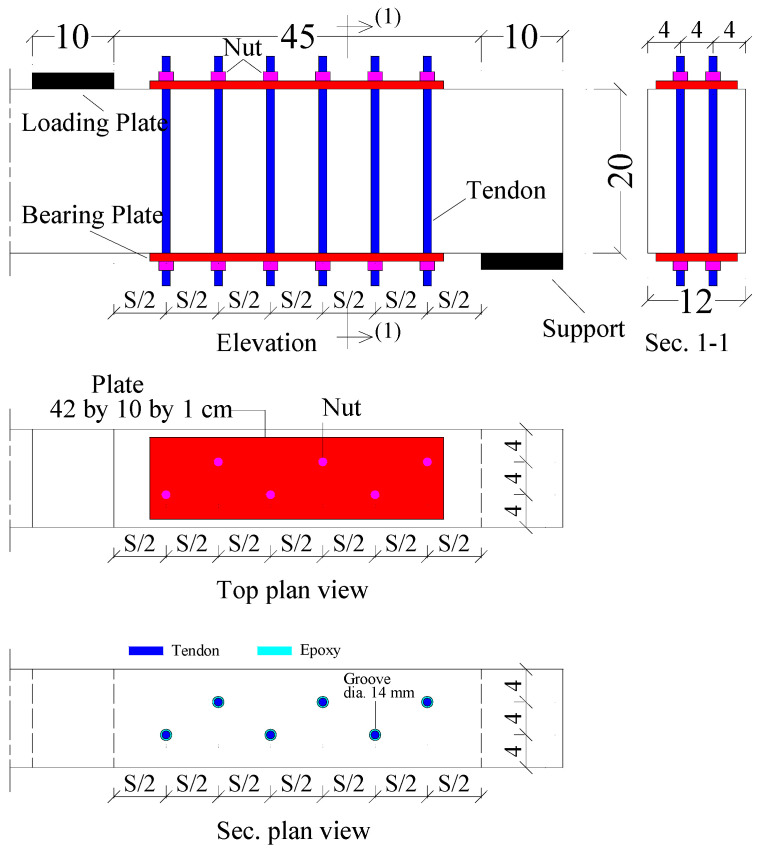
Configuration of internal prestressing conducted in three beams: IP3, IP4, and IP5 (cm).

**Figure 7:**
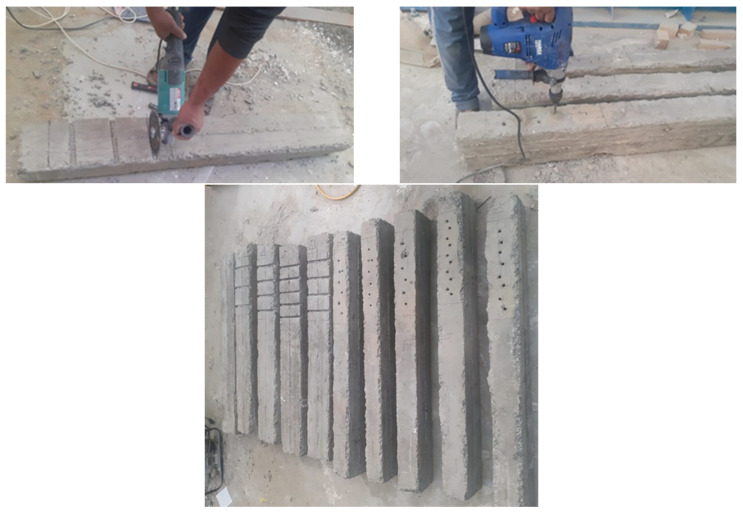
Experimental images of strengthening process.

Internal prestressing is seen in three beams (IP3, IP4, and IP5) in Figure 6. The whole height of the beam has holes punched inside of it. After thorough cleaning, epoxy is poured into the holes. Once the first prestressing force (*P_i_*) reaches the necessary amount, the strengthening rods are inserted into the holes, the steel plates are inserted within the rods, and the nuts are tightened. There were 3, 4, and 5 prestressing pair tendons in each of the beams IP3, IP4, and IP5 (Table 2). Furthermore, in the beams IP3, IP4, and IP5, the spacing (*S*) between the prestressing pair tendons was 128, 100, and 82 mm, respectively. And 1.02, 1.30, and 1.60%, respectively, were the µs values for the IP3, IP4, and IP5 beams. Three pairs of steel threaded rods were put into the beam IE3, although prestressing was not used. In contrast, the beam IE3* had three pairs of steel threaded rods put within it before the test, but they were withdrawn after the prestressing force (*P_i_* = 7.6 kN) was applied. Figure 7 shows some photos of the strengthening process.

## 3. Results and Discussion

### 3.1. Failures

**Figure 8:**
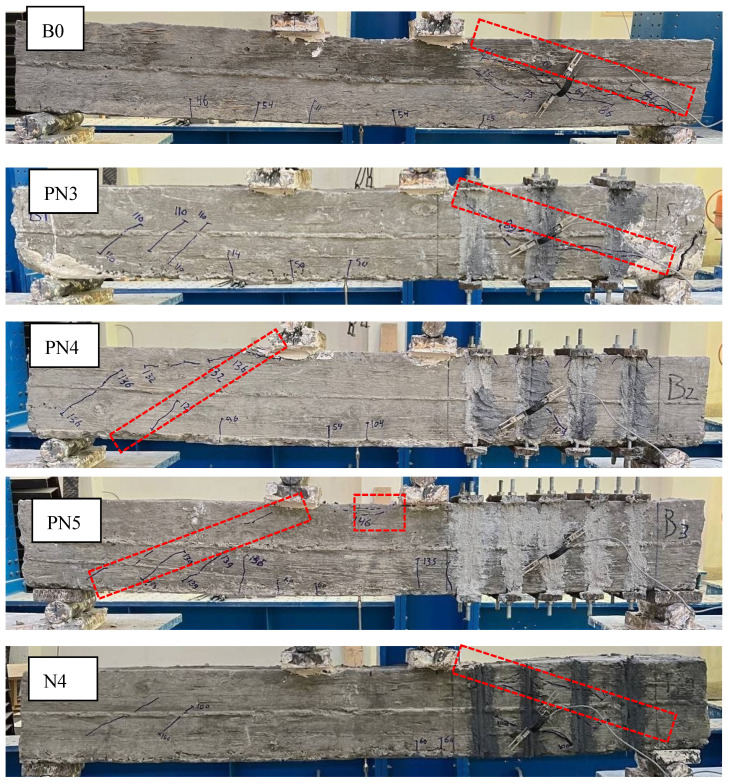

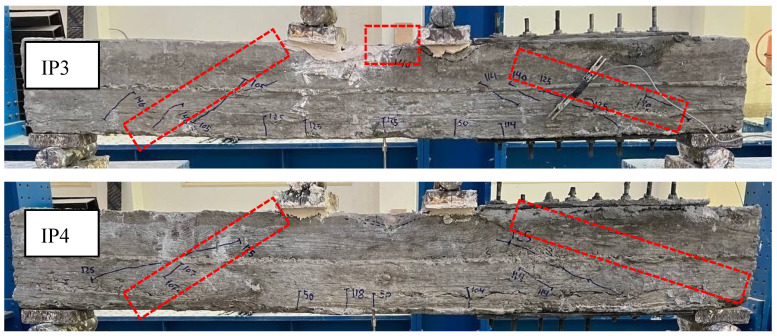
Collapses of tested beams.

The failure location and mode of tested beams are illustrated in Table 3 and Figure 8. As designed, all beams showed shear failure due to a diagonal crack starting from the support and ending at the loading point, which happened at the strengthened or/and unstrengthened shear span of the beam. The locations of shear failure were marked by red rectangles in Figure 8. Despite conducting shear strengthening at the right shear span (the strengthened part), six strengthened beams plus the control B0 failed at the right shear span due to the shear reinforcement ratio in this part being less than the shear reinforcement ratio in the left shear part (*µ_si_* = 2.08%). In the remaining strengthened beams (i.e., PN4, PN5, and IP5), strengthening by using dense reinforcement in the right shear span transferred shear failure into the left shear span (the unstrengthened part). In the beam PN5 with *µ_s_* = 1.45%, concrete crushing at the compression zone between two loading points occurred besides shear failure. In all beams, debonding at the strengthening rod–epoxy interface did not happen. Additionally, partial debonding at the concrete–epoxy interface occurred only in the beam N4 which had NSM rods glued on the beam sides. The failure modes of the beams in this study are similar with those studied in previous work [38].

### 3.2. First Crack Load

The first shear crack load (Ps) of the strengthened shear span was observed for all beams and is listed in Table 4. The Ps of the unstrengthened control beam B0 was 61 kN which was the smallest value compared to all strengthened beams, showing the success of the strengthening methods in improving the cracking load (Ps). It was seen that as the vertical strengthening rods number increased, the Ps delayed and improved more. This happened because the additional strengthening rods improved the shear strength of the concrete due to concrete confinement resulting from the strengthening rods. The beams PN4 and IP4, including four pair of steel rods, achieved the largest Ps (130 kN) compared to all beams except two, PN5 and IP5. When five pairs of steel rods were inserted into the beams PN5 and IP5, the shear cracks did not appear in the strengthened shear span. In G1, using three and four pairs of PNSM rods in PN3 and PN4 increased the Ps by 46 and 111.5%, respectively, relative to B0. The Ps of the beam N4 was 64% higher than that of the control B0, showing that using the NSM method clearly enhanced the cracking load as has happened in previous work [38]. In G3, using three and four pairs of internal prestressing rods in IP3 and IP4 increased the Ps by 105 and 113%, respectively, relative to B0. In G4, it was noticed that applying embedding rods inside the beam web also significantly improved the cracking load by around 64%. The Ps of PN4 was 29% higher than that of the beam N4 showing generating prestressing force in steel tendons enhanced clearly the cracking load. Samples of G6 confirmed that the prestressing position affected Ps, where the internal was better than the external case.

### 3.3. Load–Deflection Relationships

**Figure 9:**
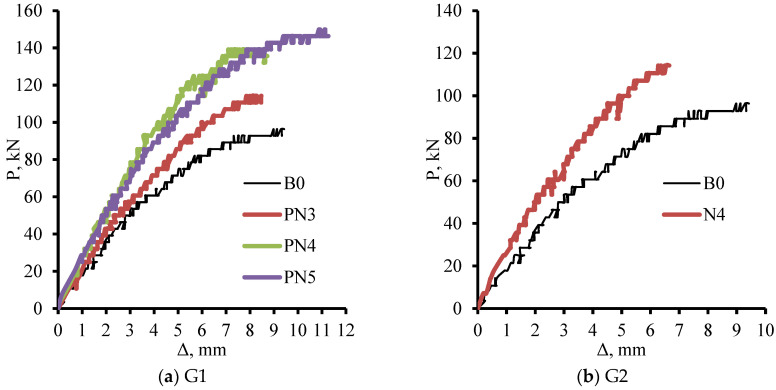

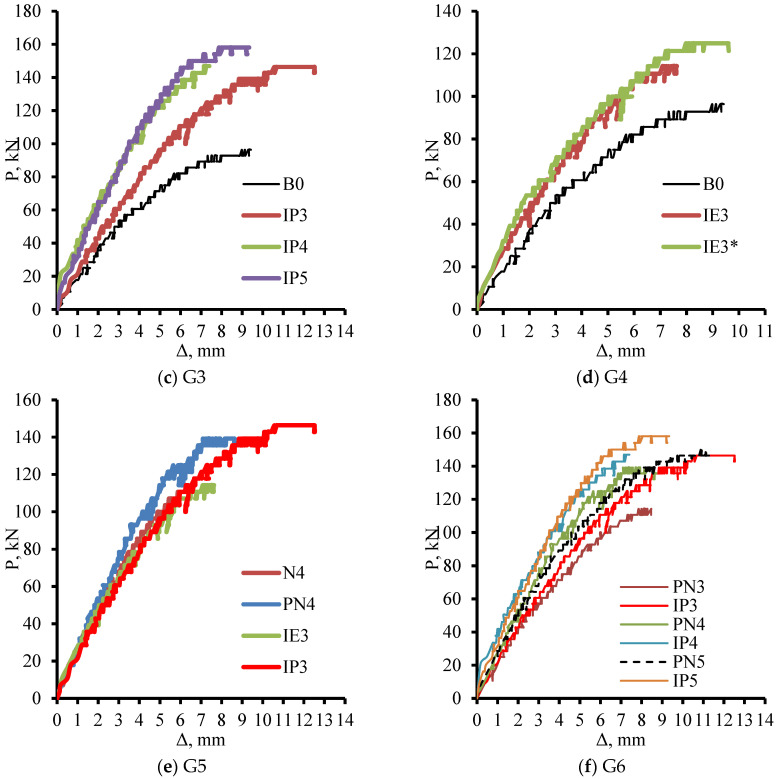
Load–mid span deflection relationships of all tested beams.

Figure 9 shows the load–mid span deflection (P-Δ) curves of all tested beams. All of the beams generally displayed nonlinear interactions. The load–deflection curve was linear from the beginning loads to about 30% of the total, and then it became nonlinear. Initially, there was a noticeable rise in the resisted load and a modest increase in the deflection rate. The deflection and the resisted load rates started to almost equalize as the loading increased. Following 70–90% of each beam’s peak load, the resisted load rose somewhat and the deflection rate was noticeably higher. In some beams, the load remained constant at its greatest value, indicating a kind of ductile behavior. In G1, which examined the impact of prestressing NSM rods, it was observed that the PNSM scheme outperformed the control beam (B0) in a pronounced way. The load of the reinforced beams (PN3, PN4, and PN5) was greater than in B0 across all places at the same degree of deformation. NSM rods were found to have significantly outperformed the control beam (B0) in the group G2. The behavior improved overall because of the additional vertical steel rods that were bonded at the beam sides at the shear span. This raised the shear reinforcement ratio.

In the group G3, the general relations over the beam B0 are made evident by using internal prestressing embedded rods in the beam web. The improvements halted in the beam IP4 when there were four rod pairs total. Stated differently, the approximate curves for IP4 (four pairs) and IP5 (five pairs) were similar. In G4, the prestressing force generated in the embedded rods did not impact overall behavior, but utilizing three pairs of rods embedded in the beam web greatly boosted the P-Δ curves related to the beam B0. Additionally, the approximate curves for IE3 with no prestressing force and IE3* with a prestressing force are the same. The G5 samples examined the impact of applying prestressing force in the context of either internal (IE3 and IP3) or exterior (N4 and PN4) strengthening. Sample PN4’s curve was marginally higher than sample N4’s, particularly in the last phases of loading. The comparison between the beam IE3 and the beam IP3 yielded the same results. Although the peak load of the beam IP3 was found to be higher than that of the beam IE3, the overall behavior remained unaffected by the presence of the prestressing force. The impact of positional prestressing strengthening in cases of internal (IP3, IP4, and IP5) or external (PN3, PN4, and PN5) strengthening was examined in G6 samples. Upon comparing the two curves of the beams PN3 and IP3, it became evident that the internal prestressing procedure had significantly enhanced the P-Δ relation. Additionally, it was observed from the curves of other beams with four and five pairs of rods that the internal prestressing performed better than the exterior prestressing due to the larger amount of concrete surrounding the inserted rods.

### 3.4. Ultimate Load Capacity

Every beam’s ultimate load (Pu) was measured and is reported in Table 5. In general, the unstrengthened control beam B0’s Pu was 92.8 kN, the lowest figure among all strengthened beams, demonstrating the effectiveness of strengthening techniques in raising Pu. On the other hand, the beam IP5, which was internally strengthened with five pairs of rods, was able to reach the greatest shear reinforcement ratio ($\mu_{s}$ = 1.6%) and therefore a heightened Pu (158.1 kN). In general, it was seen that the Pu improved more than the control beam as the number of vertical strengthening rods increased, whether they were installed internally or externally. This occurred as a result of the concrete being confined by the extra reinforcing rods, which increased the concrete’s shear strength. When five pairs of steel rods were inserted into the beams PN5 and IP5, the increase in the ratio of the shear capacity reached 57.8% and 70.4%, respectively, over the control B0. In G1, compared to B0, Pu rose by 19.3% and 46.1%, respectively, when three and four pairs of PNSM rods were used in PN3 and PN4. The Pu of the beam N4, which included four pairs of NSM rods, was 19.3% higher than that of the control B0, indicating a considerable improvement in shear capacity through the use of NSM technology. Therefore, adding external vertical NSM rods as extra shear reinforcement improves the ultimate load when prestressing force is applied to G1 samples or when using the NSM method in G2 samples.

**Figure 10:**
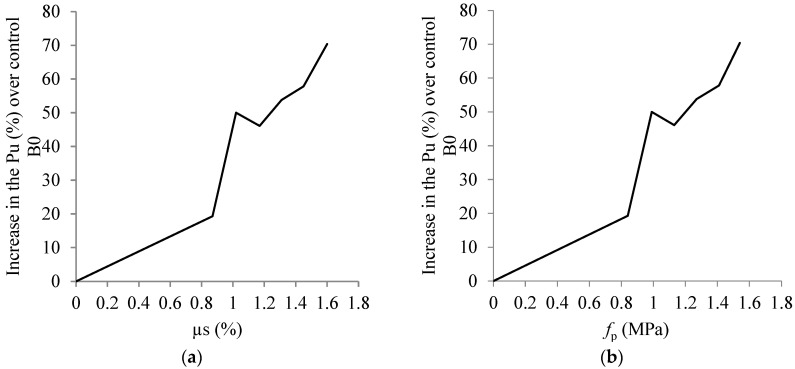
Impact of *µ_s_* and *f_p_* on the shear strength enhancement of the tested beams. (**a**) Additional shear reinforcement ratio, $\mu_{s}$. (**b**) Prestressing stress, $f_{p}$ (MPa).

In G3, the Pu rose by 50, 53, and 70%, respectively, when three, four, and five pairs of internal prestressing rods were used in IP3, IP4, and IP5, in comparison to B0. Comparable to the group G1 samples, the Pu exhibited a noticeable enhancement with the installation of internal prestressing rods, hence augmenting the shear reinforcement. Figure 10 depicts the link between rising Pu and shear reinforcement using samples from groups G1 and G3. Furthermore, a correlation was seen between the prestressing stress and the increase in Pu. The $\mu_{s}$ and $f_{p}$ had a significant impact on the tested beams’ increased shear strength. The Pu develops quickly as the $\mu_{s}$ and the $f_{p}$ rise.

It was observed in G4 samples that adding embedding rods inside the beam web greatly increased the Pu by around 30%. When three pairs of rods were installed in the beam IE3, the Pu was rose by 23.2%. When an additional prestressing force of 7.6 kN was generated in each rod then removed before the test, the Pu was rose by 34.6%. Showing positive effect of prestressing force occurred because it improved performance the shear reinforcement and delayed tension stresses inside the vertical steel rod. Based on results of G5, the Pu of PN4 was 22% more than that of the beam N4, indicating a considerable enhancement of the peak load through the generation of prestressing force in steel tendons. Also, the Pu of IP3 was 21.8% more than that of the beam IE3. It was observed that the production of prestressing force in steel tendons greatly increased the Pu when three or four pairs of inserted rods were used, as well as when strengthening was positioned both internally and externally.

G6 samples confirmed that the prestressing location had an impact on the Pu, with the internal case being superior to the external one. The Pu of IP3 that internally strengthened was 25.7% more than that of the beam PN3 that externally strengthened. Interestingly, three pairs of rods were used to reinforce these two beams, resulting in a shear reinforcement ratio (*µ_s_*) between 0.9 and 1.02%. It was observed that when the $\mu_{s}$ rose by approximately 1.0% in the beams PN4, PN5, IP4, and IP5, the prestressing position had a minor effect on the Pu.

### 3.5. Ultimate Deflection

The measurement of ultimate deflection (Δu) at the P-Δ curve’s peak is presented in Table 5. Apart from the ultimate shear capacity, the unstrengthened control beam B0 generally obtained the lowest deflection of all the reinforced beams. This shows that strengthening approaches are efficient in improving deformation. However, the beam IP5, which had five pairs of rods strengthening it inside, managed to achieve a heightened Δu (10.75 mm). Regardless of whether the vertical strengthening rods were mounted inside or externally, it was often seen that Δu improved more than in the control beam as the number of them rose. In G1, Δu increased by 2.4%, 2.7%, and 38.4%, in that order, when three, four, and five pairs of PNSM rods were employed in PN3, PN4, and PN5, in contrast to B0. Thus, when prestressing force is applied to G1 samples, the final deflection is improved by the addition of external vertical NSM rods as additional shear reinforcement. The beam N4, which included four pairs of NSM rods, had a Δu that was 18.7% lower than the control B0’s, suggesting that using NSM technology had a detrimental effect on deformation.

When three, four, and five pairs of internal prestressing rods were employed in IP3, IP4, and IP5, respectively, Δu increased by 31, −12.5, and 12.7%, relative to B0. The addition of embedding rods inside the beam web was seen to raise the Δu by approximately 18% in G4 samples. According to the G5 data, the Δu of PN4 was 26% more than that of the beam N4, showing a significant increase in peak load due to the prestressing force created in the steel tendons. Furthermore, IP3’s Δu was 41.3% greater than IE3’s. It was shown that when three or four pairs of inserted rods were employed, and when strengthening was positioned both internally and externally, the formation of prestressing force in steel tendons significantly enhanced Δu. The internal case outperformed the exterior case in the G6 samples, indicating that the prestressing site affected the Δu. The beam PN3, which was externally reinforced, had a 28% lower Δu than IP3, which was internally strengthened. It was shown that the prestressing location had an adverse influence on Δu when the µs increased by around 1.0% in the beams PN4, PN5, IP4, and IP5.

### 3.6. Rigidity

The measurement of the initial slope (k) at the P-Δ curve’s peak is presented in Table 5. To make a fair comparison between the samples, k was estimated at a specific point which was 50% of the ultimate load (P0.5) and corresponding deflection (Δ0.5). This ultimate load was divided by corresponding deflection to estimate k. One of the stiffness indices is k. In addition to the ultimate load capacity, it is crucial to investigate how the parameters that are given affect the stiffness of the strengthened beams. It is appropriate to say that the strengthening implemented in the current study improved both the ultimate load and stiffness. Of all the strengthened beams, the unstrengthened control beam B0 typically had the lowest stiffness (k = 17.7 kN/mm). This demonstrates the effectiveness of strengthening techniques in increasing stiffness. Due to having the most strengthening rods, the beam IP5 was still able to reach the maximum level of stiffness (k = 32.7 kN/mm). It was frequently observed that as the number of vertical strengthening rods increased, k improved more than in the control beam, regardless of whether they were positioned inside or externally. Compared to B0, when three, four, and five pairs of PNSM rods were used in PN3, PN4, and PN5, k rose by 3.8%, 36.2%, and 36.4%, in that sequence, in G1. Thus, by adding external vertical NSM rods as extra shear reinforcement, k is enhanced when prestressing force is applied to G1 samples. The beam N4, which featured four pairs of NSM rods, also had a k that was 27.6% higher than the control B0’s, indicating that the stiffness was clearly affected by the use of NSM technology.

Based on the G3 samples, the k rose by 20, 82, and 84.4% in comparison to B0 when three, four, and five pairs of internal prestressing rods were used in IP3, IP4, and IP5, respectively. The value of k was found to be 21.3% higher than that of the beam B0 when embedding rods were added to the IE3 beam’s web. The enhanced ratio in k reached 48.8% when tension force was supplied to these embedded rods in the beam IE3*, demonstrating that the tension force created in the embedded rods significantly enhanced k more than the absence of tension force.

According to the G5 data, the k of PN4 was 6.8% higher than that of the beam N4, showing a slight increase in rigidity due to the prestressing force created in the steel tendons. In the G6 samples, the internal case performed better than the external case, suggesting that the prestressing location had an impact on k. In comparison to IP3, which was internally reinforced, the beam PN3, which was externally reinforced, had a 15.7% lower k. Additionally, compared to the beams PN4 and PN5, which were strengthened externally, the k of the beams IP4 and IP5, which were strengthened inside, improved by 34% and 35%, respectively.

## 4. Proposed Formula for Predicting Shear Capacity

The nominal shear resistance ($V_{c}$) of the unstrengthened control beam (B0) was estimated in this section using the ACI [54] suggested formulae. A few changes were then made to anticipate the $V_{c}$ of the present strengthened beams. Using Equation (1), the $V_{c}$ that the concrete delivered was determined. The influence of the shear span to beam depth ratio (a/d) on the nominal shear strength of RC beams was investigated by Kani [55]. This section’s computation process was split into two phases. The ACI equation is applied to the control beam B0 in the first instance, and a formula is proposed in the second to forecast the shear capacity of all beams taking into account the influence of prestressing forces and the strengthening rods put into the beams. Initially, the control beam (B0)’s theoretical shear strength was calculated as follows:

(4) $$V_{c}=\beta \sqrt{f_{c}}bd=0.33\sqrt{20}*120*168=29.75\;kN$$

where $f_{c}$ is the compression property of a standard cylinder. $f_{c}$ was calculated by dividing the cubic compression property of the current concrete mix that resulted in the experiments by 1.25. *b* is the beam breadth (120 mm), *d* is the effective depth of the beam section (168 mm), and according to [55], it was obtained that *β* = 0.33 at a/d equals 3.2 in the current examined beams.

Equation (5) was used to determine the nominal shear resistance ($V_{s}$) supplied by the inner stirrups at strengthened shear span.

(5) $$V_{s}=\frac{A_{v}f_{y}d}{S}=\frac{2*28.26*251*168}{167}=14.27\;kN$$

where $f_{y}$ is yield strength of stirrups rebars (251 MPa), *A_v_* is the area of inner shear reinforcement within spacing *S* at strengthened shear span (right part).

The nominal shear strength ($V_{n}$) of unstrengthened control beam (B0) was estimated by the following equation:

(6) $$V_{n}=V_{c}+V_{s}=44.02\;kN$$

One may estimate the shear strength ($V_{T}$) of the beam (B0) based on the test results by dividing the ultimate load (Pu) by two. It was discovered that the beam B0’s $V_{T}$ was 46.4 kN. It was observed that the experimental and anticipated shear strengths of B0 are quite similar.

The modification factor ($\delta_{c}$) of Egyptian code [56] was used to estimate the nominal shear strength ($V_{c}$) of the current strengthened beams. When the concrete is subjected to compression force (Pu) acting on a specific area ($A_{c}$), the shear strength of the concrete will improve by a multiple of the factor $\delta_{c}$. The nominal shear strength ($V_{c}$) can be estimated from Equation (7).

(7) $$V_{c}=\delta_{c}\beta \sqrt{f_{c}}bd$$

(8) $$\delta_{c}=1+0.07\frac{P_{u}}{A_{c}}$$

where the term $\frac{P_{u}}{A_{c}}$ was replaced by the compression stress of prestressing force ($f_{p}$) in the current study. The factor $\delta_{c}$ must not be more than 1.5 [56].

The nominal shear strength ($V_{ss}$) provided by the additional strengthening rods was estimated using the following equation.

(9) $$V_{ss}=\frac{A_{vs}f_{ys}d}{S_{s}}$$

where $f_{ys}$ is the specified yield strength of the additional strengthening rods (390 MPa), and $A_{vs}$ is the area of strengthening rods within spacing $S_{s}$ at a strengthened shear span (two 10 mm threaded rods).

The nominal shear strength ($V_{n}$) of all tested beams was estimated by the following equation:

(10) $$V_{n}=V_{c}+V_{s}+\psi V_{ss}$$

where Ψ is the strength-reduction factor of the additional strengthening rods and is taken as 0.25. This coefficient was deduced after many mathematical attempts in order to achieve accuracy in predicting the new relationship.

Table 6 presents and computes the estimated theoretical shear strength ($V_{n}$) of all tested beams using the suggested equation. A comparison between the shear strength ($V_{T}$) experimental data and the computation outcomes of the suggested equation was conducted. The last column of Table 6 displays the theoretical-to-experimental shear strength ratio, with an average value of 1.05. The coefficient of variation (COV) and standard deviation (SD), which showed extremely low values of 0.0803 and 0.0843, respectively, demonstrate this improved agreement. Additionally, the examination showed that the outcomes derived from the suggested formula are conservative, show enough precision, and correspond favorably with the experimental data.

## 5. Conclusions

In this work, prestressing and near-surface mounted (NSM) rods placed both internally and externally in the beam web are used to shear-strengthen reinforced concrete (RC) beams which contributed to mitigating the risk severity of these RC beams. Ten RC beams with the same internal reinforcement and dimensions were subjected to flexure testing; nine of them exhibited strengthening, and one did not. The beams were intended to fail in shear collapse at a shear span that is supported by various means both internally and externally. Near-surface mounted (NSM), exterior prestressing NSM (PNSM), internal prestressing (IP) within the beam body, and internal embedment (IE) inside the beam without prestressing are the strengthening techniques that were used. In PNSM, additional shear reinforcement ratios (µs) of 0.87%, 1.17%, and 1.45% occurred, whereas in the IP method, they were 1.02%, 1.31%, and 1.6%. The µs was 1.02% and 1.17% in the IE and NSM techniques, respectively. In PNSM, the beam’s sides were cut with slots, which were then filled with epoxy and the rods placed within. The rods were then tightened to the required level. With the exception of pre-tensioning the rods, an identical process was conducted using the NSM approach. In IP, holes were bored into the web of the beam, filled with epoxy, and then rods were inserted. After that, the rods were tightened to the necessary degree. Using the IE technique, the same procedure of the IP method was carried out except that pre-tensioning the rods was not conducted. The structural behavior of strengthened beams in terms of failures, cracking load, ultimate load, load–deflection response, ultimate deflection, and stiffness was studied. The findings of the results are shown below:

(1) Debonding at the strengthening rod–epoxy contact did not occur in any of the beams. The shear behavior of RC beams was successfully improved by strengthening techniques. Shear failure moved to the other side of the beam when a high ratio of more than 1% extra shear reinforcement was employed.
(2) Referring to the control beam, the first crack load was enhanced by 46% and 111.5%, respectively, by using three and four pairs of PNSM rods, *µ_s_* = 1.17% and 1.45%. When four pairs of NSM rods, *µ_s_* = 1.17%, were placed, the first crack load improved by 64% compared to the control.
(3) The installation of five pairs of PNSM rods (*µ_s_* = 1.45%) and five pairs of IP rods (*µ_s_* = 1.6%) enhanced the shear capacity of the beams by 57.8% and 70.4%, respectively.
(4) The beam with four pairs of NSM rods (*µ_s_* = 1.17%) had a shear capacity that was 19.3% higher than the control.
(5) As the prestressing level and further shear reinforcement increase, the shear capacity of strengthened beams grows rapidly.
(6) The installation of three pairs of IE rods (*µ_s_* = 1.02%) resulted in a 23.2% increase in shear capacity while generating an additional prestressing force of 7.6 kN in each rod, then removed before the test, increased the shear capacity by 34.6%.
(7) The shear capacity was affected by the prestressing site, with the internal case outperforming the external one.
(8) The curvature of the beams can be effectively improved by strengthening techniques. The final deflection of the beams was improved by installing five pairs of PNSM rods and five pairs of IP rods, by 38.4% and 12.7%, respectively.
(9) When three, four, or five pairs of internal prestressing rods were added, the strengthened beams’ stiffness increased by 20, 82, and 84.4% relative to the control, respectively.
(10) The shear capacity of an unstrengthened beam is predicted using the ACI equation, and a formula is suggested to estimate the shear capacity of all beams while accounting for the impact of prestressing forces and strengthening rods inserted into the beams.

There are still many variables that can be studied in the future on this topic, such as the effect of the prestressing level on strengthening bars, the effect of the diameter on strengthening bars, the spacing impact on strengthening bars, and the effect of different patterns of bars on the shear capacity of RC beams.

## Figures and Tables

**Table 1:** Mixture matrix (kg/m^3^).

Basalt	Sand	Cement	Water
1290	650	300	150

**Table 2:** Matrix of strengthened beams.

Beam	N	*S* (mm)	Strengthening Positioning	Strengthening Method	*P_i_* (kN)	*f_p_* (MPa)	*µ_s_* (%)
B0	N/A	N/A	N/A	N/A	N/A	N/A	N/A
PN3	3	150	External	PNSM	7.6	0.84	0.87
PN4	4	112	External	PNSM	7.6	1.13	1.17
PN5	5	90	External	PNSM	7.6	1.41	1.45
N4	4	112	External	NSM	0	0.00	1.17
IP3	3	128	Internal	IP	7.6	0.99	1.02
IP4	4	100	Internal	IP	7.6	1.27	1.31
IP5	5	82	Internal	IP	7.6	1.54	1.60
IE3	3	128	Internal	IE	0	0.00	1.02
IE3*	3	128	Internal	IE	7.6 ^a^	0.99	1.02

N is the number of tendon pairs. *S* is spacing between each tendon pair. *P_i_* is the initial prestressing force applied in each tendon. *f_p_* is the prestressing stress acting on the beam web. *µ_s_* is the added shear reinforcement ratio due to strengthening. PNSM means prestressing near-surface mounted. IP means internally prestressed. IE means internally embedded in the beam web. ^a^ denotes that this tension force was removed before the test.

**Table 3:** Failures of tested beams.

Beam	B0	PN3	PN4	PN5	N4	IP3	IP4	IP5	IE3	IE3*
Failure type	SF	SF	SF	SF-a	SF-b	SF-a	SF	SF-a	SF	SF
Failure location	SSS	SSS	USS	USS	SSS	SSS + USS	SSS + USS	USS	SSS	SSS

SF is shear failure, while a refers to the occurrence of concrete crushing at compression zone between two loading points and b refers to the occurrence of partial debonding at the concrete–epoxy interface. USS is the unstrengthened shear span (left part). SSS is the strengthened shear span (right part).

**Table 4:** The first shear crack load of the tested beams.

Group	Variable	Beam	Ps	Gain in Ps (%)
G1	PNSM	B0	61	0.00
		PN3	89	45.90
		PN4	129	111.48
		PN5	NO	N/A
G2	NSM	B0	61	0.00
		N4	100	63.93
G3	Internal prestressing	B0	61	0.00
		IP3	125	104.92
		IP4	130	113.11
		IP5	NO	N/A
G4	Applying embedding	B0	61	0.00
		IE3	100	63.93
		IE3*	100	63.93
G5	Applying prestressing	N4	100	0.00
		PN4	129	29.00
		IE3	100	0.00
		IP3	125	25.00
G6	Prestressing position	PN3	89	0.00
		IP3	125	40.45
		PN4	129	0.00
		IP4	130	0.78
		PN5	NO	N/A
		IP5	NO	N/A

**Table 5:** Analysis of the results.

Group	Variable	Beam	Pu (kN)	Gain in Pu (%)	Δu (mm)	Gain in Δu (%)	P_0.5 (kN)_	Δ_0.5_ (mm)	k = P_0.5_/Δ_0.5_ (kN/mm)	Gain in k (%)
G1	PNSM	B0	92.8	0.0	7.77	0.0	46.4	2.62	17.7	0.0
		PN3	110.7	19.3	7.96	2.4	55.35	3.01	18.4	3.8
		PN4	135.6	46.1	7.98	2.7	67.8	2.81	24.1	36.2
		PN5	146.4	57.8	10.75	38.4	73.2	3.03	24.2	36.4
G2	NSM	B0	92.8	0.0	7.77	0.0	46.4	2.62	17.7	0.0
		N4	110.7	19.3	6.32	−18.7	55.35	2.45	22.6	27.6
G3	Internal prestressing	B0	92.8	0.0	7.77	0.0	46.4	2.62	17.7	0.0
		IP3	139.2	50.0	10.2	31.3	69.6	3.27	21.3	20.2
		IP4	142.7	53.8	6.8	−12.5	71.35	2.21	32.3	82.3
		IP5	158.1	70.4	8.76	12.7	79.05	2.42	32.7	84.4
G4	Applying embedding	B0	92.8	0.0	7.77	0.0	46.4	2.62	17.7	0.0
		IE3	114.3	23.2	7.22	−7.1	57.15	2.66	21.5	21.3
		IE3*	124.9	34.6	9.21	18.5	62.45	2.37	26.4	48.8
G5	Applying prestressing	N4	110.7	0.0	6.32	0.0	55.35	2.45	22.6	0.0
		PN4	135.6	22.5	7.98	26.3	67.8	2.81	24.1	6.8
		IE3	114.3	0.0	7.22	0.0	57.15	2.66	21.5	0.0
		IP3	139.2	21.8	10.2	41.3	69.6	3.27	21.3	−0.9
G6	Prestressing position	PN3	110.7	0.0	7.96	0.0	55.35	3.01	18.4	0.0
		IP3	139.2	25.7	10.2	28.1	69.6	3.27	21.3	15.7
		PN4	135.6	0.0	7.98	0.0	67.8	2.81	24.1	0.0
		IP4	142.7	5.2	6.8	−14.8	71.35	2.21	32.3	34.0
		PN5	146.4	0.0	10.75	0.0	73.2	3.03	24.2	0.0
		IP5	158.1	8.0	8.76	−18.5	79.05	2.42	32.7	35.0

**Table 6:** The predicted shear strength versus experimental results.

Beam	*S_s_* (mm)	$f_{p}$ (MPa)	$\delta_{c}$	$V_{n}$ (kN)	$V_{s}$ (kN)	$V_{ss}$ (kN)	ψ	$V_{n}$ (kN)	$V_{T}$ (kN)	$\frac{V_{n}}{V_{T}}$
B0	0	0	1.00	29.75	14.27	0.00	0.00	44.02	46.40	0.95
PN3	150	0.84	1.06	31.50	14.27	68.58	0.25	62.91	55.35	1.14
PN4	112	1.13	1.08	32.10	14.27	91.85	0.25	69.33	67.80	1.02
PN5	90	1.41	1.10	32.69	14.27	114.30	0.25	75.53	73.20	1.03
N4	112	0	1.00	29.75	14.27	91.85	0.25	66.98	55.35	1.21
IP3	128	0.99	1.07	31.81	14.27	80.36	0.25	66.17	69.60	0.95
IP4	100	1.27	1.09	32.39	14.27	102.87	0.25	72.38	71.35	1.01
IP5	82	1.54	1.11	32.96	14.27	125.45	0.25	78.59	79.05	0.99
IE3	128	0	1.00	29.75	14.27	80.36	0.25	64.11	57.15	1.12
IE3*	128	0.99	1.07	31.81	14.27	80.36	0.25	66.17	62.45	1.06
Average										1.05
SD										0.0843
COV										0.0803

## Data Availability

The original contributions presented in the study are included in the article, further inquiries can be directed to the corresponding author.
